# Relationship Between Rod-Mediated Sensitivity, Low-Luminance Visual Acuity, and Night Vision Questionnaire in Age-Related Macular Degeneration

**DOI:** 10.1167/tvst.9.6.30

**Published:** 2020-05-28

**Authors:** Myra B. McGuinness, Rogan G. Fraser, Rose Tan, Chi D. Luu, Robyn H. Guymer

**Affiliations:** 1 Centre for Eye Research Australia, Royal Victorian Eye and Ear Hospital, East Melbourne, Victoria, Australia; 2 Department of Surgery (Ophthalmology), The University of Melbourne, East Melbourne, Victoria, Australia

**Keywords:** age-related macular degeneration, rods, dark adaptation, visual field, night vision

## Abstract

**Purpose:**

To quantify the association between dark adaptation parameters and other clinical measures of visual function among people with and without early and intermediate age-related macular degeneration (AMD).

**Methods:**

In this cross-sectional study, participants underwent multimodal imaging and visual function testing, including best-corrected visual acuity (BCVA), low-luminance visual acuity (LLVA), low-luminance deficit (LLD = BCVA – LLVA) and the 10-item Night Vision Questionnaire (NVQ-10). Dynamic and static dark-adapted chromatic perimetry (DACP) was performed. Sensitivity difference was defined as the difference in sensitivity between the 505-nm and 625-nm stimuli. Rod intercept time (RIT) was estimated as the time required to reach a threshold of −3 log candelas/meter^2^ with the 505-nm stimulus following bleaching. The magnitude of association between the DACP parameters and other clinical tests was estimated via mixed-effects regression.

**Results:**

A total of 51 participants (aged 51–88 years, 65% female, 39% with AMD) were included. RIT was found to be negatively associated with BCVA (*P* < 0.001), LLVA (*P* = 0.005), and NVQ-10 score (*P* = 0.028) but not LLD (*P* = 0.763). There was no evidence of an association between sensitivity difference and any of the clinical measures (*P* ≥ 0.081).

**Conclusions:**

Reduced rod function, as determined by RIT, was associated with lower NVQ-10 scores (designed to interrogate rod-mediated function) and with worse BCVA and LLVA (measures of cone function).

**Translational Relevance:**

Decreasing rod function maybe indicative of more generalized photoreceptor dysfunction involving cones. Further development of questionnaires to target function in scotopic conditions may provide an easier to administer test without the need to perform perimetric tests of rod function.

## Introduction

Reductions in rod-mediated visual function have been well documented as an early functional deficit in age-related macular degeneration (AMD), present even when best-corrected visual acuity (BCVA) remains unaffected.^1^^–^^6^ Indices of rod function have therefore been suggested as potential early biomarkers of disease severity in AMD and may potentially be used in clinical trials to investigate the efficacy of new interventions at early stages of disease in the absence of anatomical signs of progression to late-stage AMD.^7^^–^^9^ However, the current psychophysical measurement of rod function is time-consuming and logistically difficult among elderly patients and in clinical settings.^10^

Other more simple clinical tests of visual function have also been used to monitor functional changes in the early stages of AMD.^7^^,^^11^ Low-luminance visual acuity (LLVA) and low-luminance deficit (LLD) have shown promise as potential functional biomarkers in clinical AMD trials.^7^^,^^11^ Several studies have documented poor performance of visual tasks in low-luminance settings despite good visual acuity in the early stages of AMD.^7^^,^^11^^–^^14^ In addition, questionnaires that aim to interrogate visual function in dim or dark conditions have also been derived in an attempt to capture the difficulty experienced in the dark.^12^^,^^15^^–^^18^

However, in the early stages of AMD, it remains uncertain how well the changes in rod function, as assessed by formal perimetric testing, correlate with these other tests and low-luminance questionnaires, which also aim to capture the difficulties experienced in the early stages of disease before BCVA is affected. Therefore, the purpose of this study was to determine the association between rod-mediated sensitivity measured via dark-adapted chromatic perimetry (DACP) and each of BCVA, LLVA, LLD and the 10-item Night Vision Questionnaire (NVQ-10).

## Methods

This was a cross-sectional observational study that was approved by the Human Research and Ethics Committee of the Royal Victorian Eye and Ear Hospital. Written informed consent conforming to the tenets of the Declaration of Helsinki was acquired from all participants prior to commencing the study.

### Participants

Participants were recruited from existing prospective natural history research cohorts in the Macular Research Unit at the Centre for Eye Research Australia, Melbourne, Australia, between 2015 and 2017. Control participants were recruited from spouses, friends, and relatives of the AMD participants and from among staff.

Eligibility criteria for this analysis included age ≥50 years with BCVA of ≥60 letters (equivalent to 6/19 or 20/63). When both eyes were eligible to be included, the eye with the best BCVA was chosen as the study eye.

Participants with evidence of current or past neovascular AMD or geographic atrophy (as defined below) in either eye were excluded, as were participants with ungradable retinal images. Participants with unilateral reticular pseudodrusen (RPD) were also excluded, as RPD is known to affect dark adaptation and may be a confounding factor when investigating the relationship between uniocular rod function and NVQ-10 score (which relates to binocular vision).^19^

Exclusion criteria for both groups included people with grade 2 cataract or worse (World Health Organization [WHO] grading system), diabetic retinopathy, glaucoma, neck or spinal problems preventing completion of DACP, or medications that might affect retinal function such as hydroxychloroquine.^20^ In addition, participants with medical conditions that could affect dark adaptation (such as liver disease and renal disease) were excluded.^21^^,^^22^

### Visual Acuity

BCVA was measured at 4 meters using a modified count-letters version of the Early Treatment of Diabetic Retinopathy Study protocol.^23^ LLVA was measured under the same condition but with the addition of a 2.0 neutral density filter in front of the study eye. LLD was calculated by subtracting LLVA from BCVA.^24^ All visual acuity measures were recorded with best refractive correction.

### NVQ-10

The NVQ-10 possesses two distinct subscales to assess self-reported visual function in low luminance.^17^ The first subscale relates to car travel and has five response options ranging from *no* *difficulty at all* to *stopped* *doing because of my eyesight* as seen in Supplementary Table S1. Additional response options, *stopped* *doing for reasons other than my eyesight* and *not* *currently driving*, were treated as missing for the purposes of this analysis. The second subscale relates to how bothered participants are by their vision and has four response options ranging from *not* *at all* to *very*.

### DACP

Pupils were dilated to a minimum of 6 mm with 0.5% tropicamide (Mydriacyl; Alcon Laboratories, NSW, Australia). Sphero-cylindrical lens correction was inserted into a lens holder with the refractive correction set up for a viewing distance of 30 cm. Fixation was monitored via an infrared-activated camera throughout testing. All participants were given the same instructions prior to testing.

The Medmont DACP (Medmont International Pty Ltd, Nunawading, Australia) has two color stimuli, one at 505 nm (cyan, dynamic range of 0–75 dB) and the other at 625 nm (red, dynamic range of 0–50 dB).^25^ Stimuli of 1.73° in diameter (Goldmann size V) were presented for durations of 200 ms. Thresholds were determined using a 4-2 staircase threshold strategy.^25^

Dynamic threshold testing was conducted following 20% bleaching of the rod photopigment from a single flash of approximately 2.45 × 10^6^ scotopic candelas per meter squared (cd/m^2^) in intensity from a customized Ganzfeld stimulator (Mecablitz 45 CL-4; Metz-Werke GmbH & Co., Zirndorf, Germany).^26^ Over 30 minutes, the sensitivity to the 505-nm stimulus was measured at 14 test locations 4°, 5.7°, 8°, and 12° from the fovea.

Static threshold testing was then conducted using the 625-nm (red) stimuli, which was undertaken following a short break to avoid fatigue. Twenty-four test loci in total were located at 4°, 5.7°, 8°, 12°, 17°, and 24° from the fovea.

All testing was performed monocularly with the fellow eye occluded.

### Retinal Imaging and Classification

All participants underwent multimodal imaging (near-infrared reflectance, short-wavelength fundus autofluorescence, optical coherence tomography [Spectralis HRAþOCT; Heidelberg Engineering, Heidelberg, Germany], and color fundus photography [Canon CR6-45NM; Canon, Saitama, Japan]), which was performed following functional assessment to prevent retinal bleaching. Grading was performed by trained graders who were masked to participant characteristics.

AMD was classified according to the Beckman classification system.^27^ Control participants had no apparent aging changes (no drusen and no AMD pigmentary abnormalities) or drupelets (small drusen ≤63 µm) only.^27^ In the absence of geographic atrophy (defined within a radius of 3000 mm from the fovea, as any area >175 mm in diameter of partial or complete retinal pigment epithelium hypopigmentation with visible underlying large choroidal vessels that was either roughly round or oval and showed sharp margins on color fundus photography) or choroidal neovascularization detected on any imaging modality, participants with drusen sized 63 to <125 µm without pigmentary abnormalities were considered to have early AMD, and those with drusen >125 µm or drusen 63 to <125 µm with pigmentary abnormalities were considered to have intermediate AMD. RPD were defined as clear round or cone-shaped subretinal deposits between external limiting membrane or outer plexiform layers and retinal pigment epithelium and determined using all imaging modalities.^28^^,^^29^

### Crystalline Lens Grading

Lens status was assessed through dilated pupils using slit-lamp microscopy and graded according to the WHO classification scheme after tests of visual function had been completed.^20^

### Data Analysis

It has previously been shown that AMD status has a greater impact on rod function for loci in the central visual field than those peripherally.^19^^,^^30^ Therefore, only DACP data from the central 8° (12 loci) were analyzed (as specified a priori).

The sensitivity difference between the 505-nm and 625-nm stimuli (cyan—red sensitivity difference) was derived at each locus to enable psychophysical assessment of rod-mediated function based on the two-color perimetry principle.^19^^,^^25^ In dark-adapted healthy eyes, the sensitivity to the 505-nm stimulus is expected to be greater than that of the 625-nm stimulus. Rod cells absorb 505-nm stimuli to a greater extent than cone cells but are insensitive to the 625-nm stimulus, which is detected by cones.^6^ Therefore, the sensitivity difference becomes smaller as rod function declines in relation to cone function.

Rod intercept time (RIT) was defined as the time required following bleaching for sensitivity to recover to −3.0 log cd/m^2^ stimulus intensity for each test locus.^30^ The relationship between luminance and time has previously been modeled as a series of exponential decay functions.^31^^,^^32^ For this analysis, we used the formula previously presented by our group and derived the parameters of this model using nonlinear least squares estimation.^26^^,^^33^ RIT was then determined algebraically using these estimation parameters. The statistical computing code used to estimate the RIT is presented in the Supplementary Material. RIT could not be estimated for test loci with sensitivity better than −3 log cd/m^2^ prior to the first response to stimuli (indicating unreliable responses or incomplete bleaching) or if sensitivity did not recover to better than −3 log cd/m^2^ within 30 minutes of testing.

DACP values from each ring were compared between AMD participants and controls using Somers’ *D* statistic, accounting for within-participant correlation.

Estimates of the latent trait of visual function in low luminance were derived from NVQ-10 responses. The first two response categories for questionnaire items 1 to 4 were collapsed due to low response rates for these categories (see Supplementary Table S1). A partial-credit model was then used to estimate NVQ-10 scores due to a violation of the rating scale model assumption of step invariance.^34^

**Figure 1.**
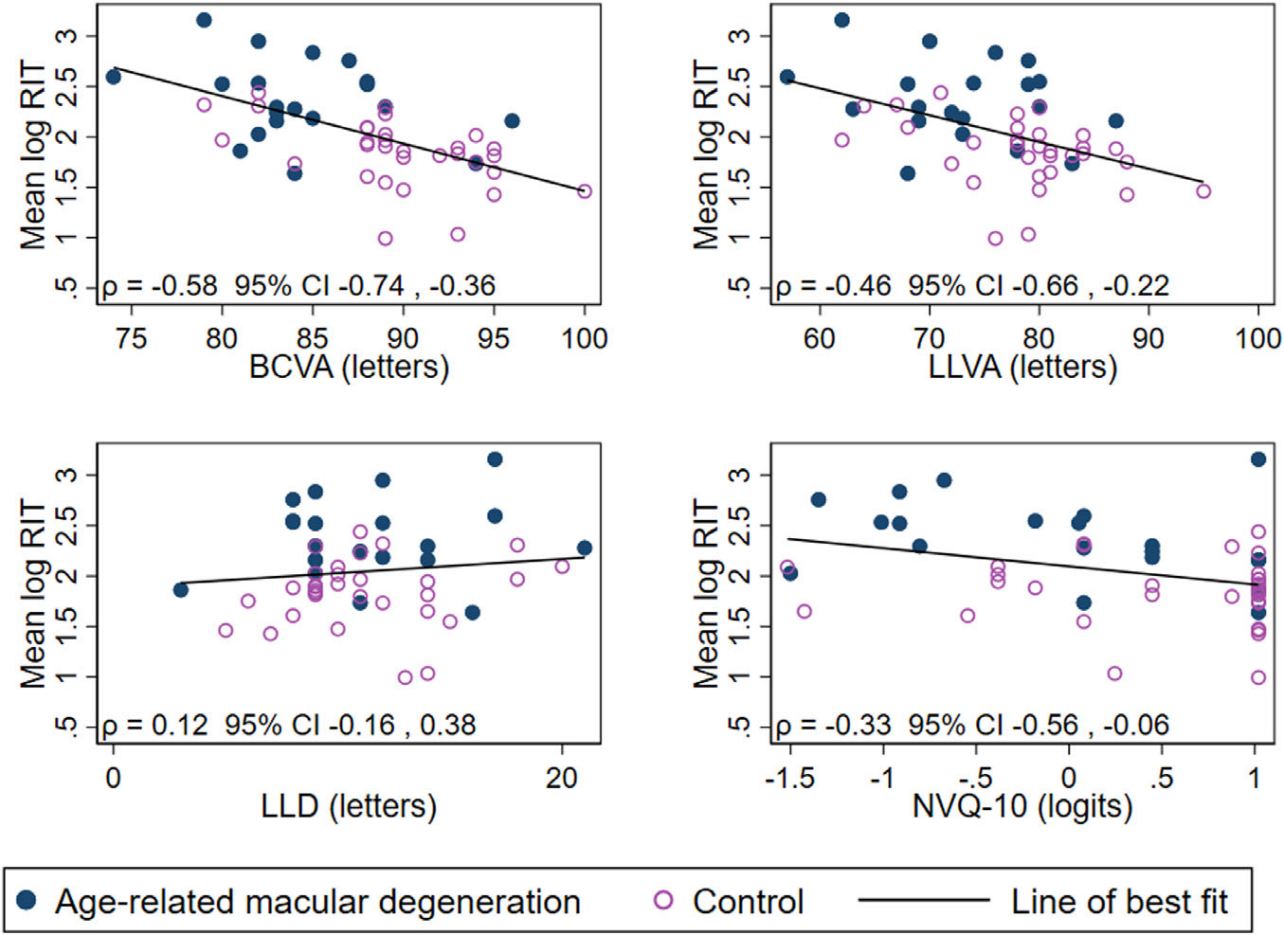
Scatterplots of the relationship between the average of the natural log of RIT and each of BCVA, LLVA, LLD, and NVQ-10 score (*n* = 51). *ρ* = Pearson's correlation coefficient for average log-RIT for each participant and each of BCVA, LLVA, LLD, and NVQ-10.

The association between RIT and NVQ-10 score, BCVA, LLVA, and LLD was found to be approximately linear after log-transformation of RIT (see Fig. 1). Therefore, the relationship between the natural logarithm of DACP parameters and each of the other tests was investigated via mixed-effects linear regression, adjusting for lens status and age (specified as potential predictors of rod function a priori) and accounting for within-person and within-ring correlation. The identity variance-covariance structure was chosen for the random effects, and the model was fit via restricted maximum likelihood estimation. This relationship was graphically inspected using scatterplots of the mean log-transformed DAPC parameters for each participant against each of the other test parameters that were presented with Pearson's correlation coefficients.

A complete case analysis was performed; that is, only participants with data on RIT, sensitivity difference, NVQ-10, and visual acuity variables were included. Statistical analyses were performed using Stata/SE 16.0 (StataCorp LLC, College Station, TX, USA).

## Results

A total of 103 participants were screened for inclusion. Data from 51 participants (50%) remained after exclusions (see Supplementary Figure S1 for participant flowchart). A summary of demographic and clinical data for included participants is presented in Table 1. Participants ranged from 51 to 88 years of age, and the majority were female (65%). Early AMD was detected in 5 participants (10%), and 15 (29%) had intermediate AMD. There were 31 age-matched control participants.

**Table 1.** Participant Demographic and Clinical Characteristics

Characteristic	AMD (*n* = 20)	Control (*n* = 31)	Total (*N* = 51)
Age, mean (SD), y	69.8 (7.5)	66.1 (9.6)	67.6 (8.9)
Sex, *n* (%)			
Male	5 (25)	13 (42)	18 (35)
Female	15 (75)	18 (58)	33 (65)
Lens status, *n* (%)^a^			
Clear	13 (65)	19 (61)	32 (63)
Intraocular lens	1 (5)	1 (3)	2 (4)
Cataract	6 (30)	11 (35)	17 (33)
NVQ-10 score (logits), median (IQR)	0.1 (−0.9, 0.7)	1.0 (0.1, 1.0)	0.4 (−0.4, 1.0)
BCVA (letters), mean (SD)^a^^,^^b^	84.4 (5.0)	89.6 (4.7)	87.6 (5.4)
LLVA (letters), mean (SD)^a^^,^^b^	73.0 (7.5)	78.5 (7.2)	76.4 (7.7)
LLD (letters), mean (SD)^a^^,^^b^	11.4 (4.1)	11.1 (3.5)	11.3 (3.7)

IQR, interquartile range.

a Values from a single eye (the study eye) per participant.

b Missing values for one control participant.

### DACP

All participants were considered reliable, with false-positive rates of less than 10%. A total of 582 loci from 51 participants were included in the analyses. RIT was estimated for all 12 loci among 42 of 51 participants (82%). Of the remaining nine participants with at least one locus for which RIT could not be estimated, six AMD participants (with bilateral RPD) had RIT estimated to be greater than 30 minutes and three control participants with loci that had reached adaptation prior to ascertainment of threshold.

Summary statistics for each parameter of interest are presented in Table 2 according to distance from fixation and AMD status. In general, there was stronger evidence for a difference between AMD and control participants when assessing loci at 4° and 5.657° from fixation (compared to loci at 8°) and when assessing RIT (compared to other DACP parameters). There was a weak correlation between RIT and sensitivity difference values (Spearman's correlation −0.16; 95% confidence interval, −0.24 to −0.08).

**Table 2.** Dark-Adapted Chromatic Perimetry Values by Distance from Fixation and Age-Related Macular Degeneration Status

	Median (Interquartile Range)			
Characteristic	Total (*N* = 51)	AMD (*n* = 20)	Control (*n* = 31)	*P* Value^a^
Cyan threshold (505 nm), dB				
All points^b^	52.0 (48.0, 56.0)	50.0 (48.0, 52.0)	52.0 (48.0, 56.0)	0.005
4°	48.0 (46.0, 52.0)	48.0 (44.0, 48.0)	49.0 (48.0, 52.0)	0.002
5.657°	52.0 (48.0, 56.0)	50.0 (48.0, 52.0)	54.0 (50.0, 56.0)	<0.001
8°	54.0 (50.0, 56.0)	52.0 (50.0, 56.0)	54.0 (50.0, 56.0)	0.158
Red threshold (625 nm), dB				
All points^b^	30.0 (28.0, 34.0)	30.0 (28.0, 32.0)	32.0 (30.0, 34.0)	0.003
4°	30.0 (26.0, 30.0)	28.0 (26.0, 30.0)	30.0 (28.0, 32.0)	0.003
5.657°	30.0 (28.0, 34.0)	30.0 (28.0, 30.0)	32.0 (30.0, 34.0)	0.002
8°	32.0 (30.0, 34.0)	30.0 (30.0, 32.0)	34.0 (30.0, 34.0)	0.005
Sensitivity difference, dB				
All points^b^	20.0 (18.0, 24.0)	20.0 (18.0, 22.0)	22.0 (18.0, 24.0)	0.204
4°	20.0 (18.0, 22.0)	20.0 (18.0, 22.0)	20.0 (18.0, 22.0)	0.381
5.657°	22.0 (20.0, 24.0)	20.0 (18.0, 22.0)	22.0 (20.0, 24.0)	0.019
8°	22.0 (20.0, 24.0)	22.0 (20.0, 24.0)	22.0 (18.0, 24.0)	0.771
Rod intercept time, min				
All points^*^	7.2 (5.9, 9.8)	9.9 (7.6, 12.8)	6.5 (5.4, 7.9)	<0.001
4°	7.0 (5.7, 9.8)	10.0 (7.8, 14.0)	6.3 (5.3, 7.8)	<0.001
5.657°	7.2 (5.8, 9.9)	9.9 (7.7, 12.7)	6.3 (5.5, 7.6)	<0.001
8°	7.4 (6.0, 9.8)	9.5 (7.5, 12.3)	6.7 (5.5, 8.0)	<0.001

*n* = number of participants, with multiple test points within each ring for each participant (528 test loci total).

a *P* values comparing AMD to control participants estimated using Somers’ *D* statistic accounting for within-participant correlation.

b Threshold values and rod intercept time values taken from all visual field loci within 8° from fixation for a single eye per participant.

### NVQ-10

Ceiling effects were observed for NVQ-10 responses, indicating that many participants reported no visual impairment in mesopic conditions (see Supplementary Table S1 for tabulation of responses to each item). Ten percent of participants did not drive a car for reasons other than poor eyesight; items 1 to 3 were therefore not applicable for these participants.

**Figure 2.**
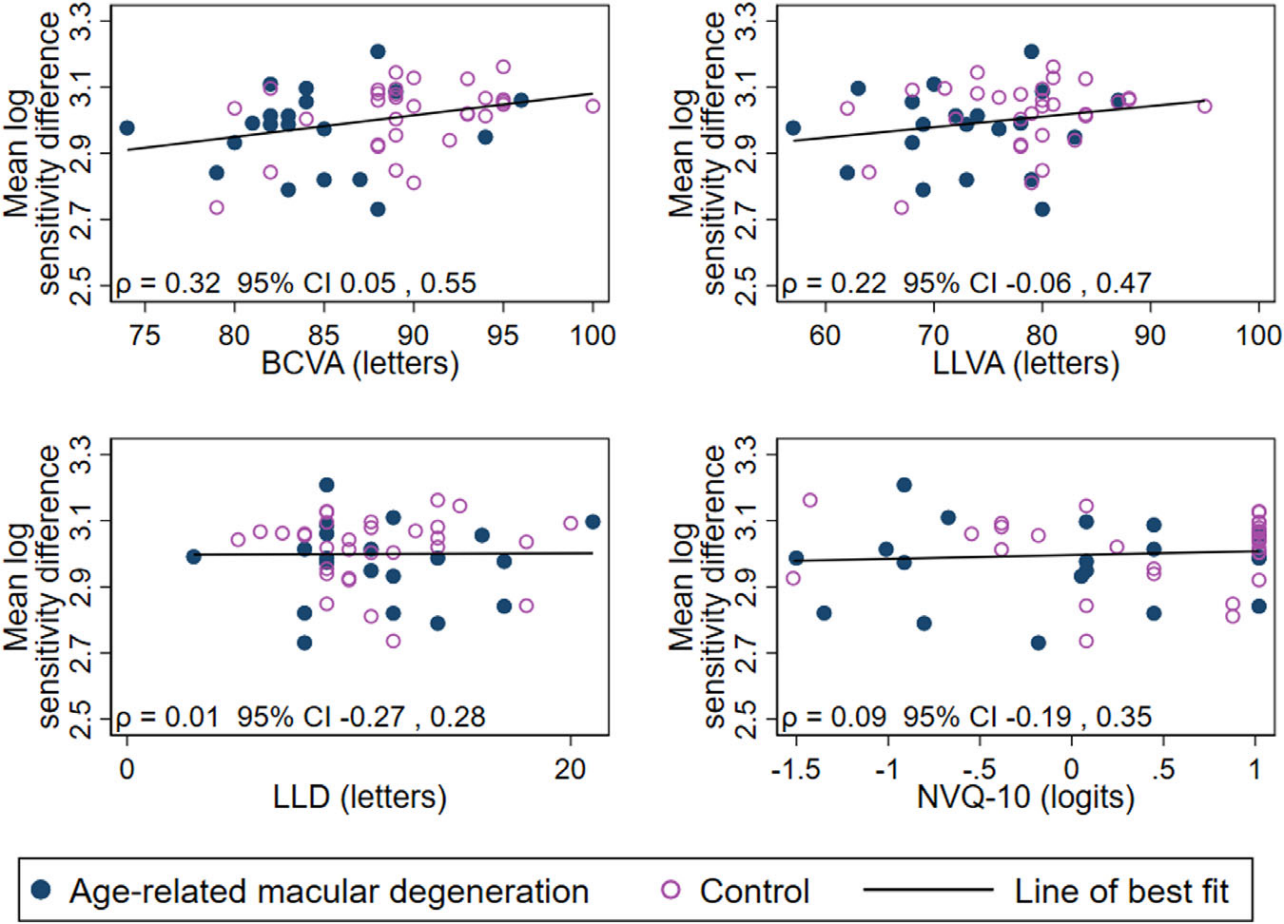
Scatterplots of the relationship between average of the natural log of sensitivity difference and each of BCVA, LLVA, LLD, and NVQ-10 score (*n* = 51). *ρ* = Pearson's correlation coefficient for average log-sensitivity difference for each participant and each of BCVA, LLVA, LLD, and NVQ-10.

There was a weak negative association between NVQ-10 score and RIT, as seen in Table 3 (age- and lens-adjusted estimates) and Figure 1 (unadjusted correlation). However, there was no evidence of an association between NVQ-10 and sensitivity difference (Fig. 2).

**Table 3.** Association Between Dark-Adapted Chromatic Perimetry Values and Other Clinical Tests (*N* = 51 Participants)

	Rod Intercept Time			Sensitivity Difference		
Characteristic	% Change	95% CI	*P* Value^a^	% Change	95% CI	*P* Value^a^
BCVA (per letter increase)	−4.2	−6.2 to −2.2	<0.001	0.5	−0.1 to 1.1	0.081
LLVA (per letter increase)	−2.2	−3.7 to −0.7	0.005	0.2	−0.2 to 0.6	0.334
LLD (per letter increase)	0.5	−2.7 to 3.9	0.763	0.2	−0.6 to 1.0	0.650
NVQ-10 score (per logit increase)	−14.7	−25.9 to −1.7	0.028	1.5	−2.2 to 5.4	0.422

CI, confidence interval.

a Estimated using mixed-effects linear regression with log-transformed values of rod intercept time or sensitivity difference, adjusted for age and lens status, with random intercepts for participant and distance from fixation.

### Measures of Visual Acuity

BCVA ranged from 74 (6/9.5 or 20/32) to 100 letters (6/6 or 20/20). RIT was estimated to decrease with increases in BCVA and LLVA (adjusted difference −4.2% and −2.2% per letter increase, respectively; see Table 3 and Fig. 1). However, no association could be found between LLD and RIT. No evidence of an association was detected between sensitivity difference and any of the visual acuity-based tests (*P* ≥ 0.081).

## Discussion

The primary aim of this study was to investigate the association between rod function (as measured via DACP) and other clinical measures of visual function. We found a negative correlation between RIT and both BCVA and LLVA as well as a weak correlation between RIT and self-reported low-luminance vision.

A strong association was observed between RIT, a test of rod-mediated function, and BCVA, a test of cone function. This implies that in the early stages of AMD, even though we find BCVA to be fairly normal, there are indications of a subtle decline of visual acuity along with the better-recognized decline in rod function. The association between the formal testing of rod function by RIT and of self-reported visual function in dim conditions by NVQ-10 was not as strong as that seen between RIT and BCVA.

### Comparison to Previous Literature

Flamendorf and coauthors^35^ also reported a moderate negative correlation between RIT (assessed at a single locus) and BCVA. A slightly stronger association between RIT (assessed at a single locus) and self-reported low-luminance vision was found among people with intermediate AMD and healthy controls by Yazdanie and coauthors.^36^ In that study, self-reported outcomes were assessed using the 32-item Low Luminance Questionnaire (LLQ-32).^12^ With more than three times the number of items than the NVQ-10, the LLQ-32 can better discriminate between levels of functional vision than the NVQ-10.

Our group has previously reported on the relationship between NVQ-10 scores and clinical measures of visual function among people with bilateral intermediate AMD.^14^ In that study, self-reported visual function was more highly correlated with LLD than with LLVA. In the present study, which included participants with early AMD and healthy controls as well as participants with intermediate AMD, we found RIT to be more concordant with LLVA than LLD. This variation may be due to different populations or may simply represent random variation. However, LLVA and LLD are known to be correlated.

### Strengths and Limitations

Dark adaptation was only measured over a period of 30 minutes, which is less than some other studies.^35^^,^^36^ Therefore, RIT could not be estimated for all loci of all participants. However, the number of participants with RIT from any locus estimated to be greater than 30 minutes was small, and all participants were able to be included in the analyses due to the statistical modeling approach (which does not require participants to have data at every visual field locus).

Reduction in cyan-red sensitivity represents decline in rod function relative to cone function. However, the sensitivity difference will underestimate the magnitude of rod function dysfunction if cone cell function is also abnormal.^6^ For this reason, participants with pathology other than AMD were excluded from this study.

The NVQ-10 was not designed using modern psychometric approaches,^17^ and newer instruments designed to capture vision-related quality of life in low-luminance settings may show greater a correlation with rod function. This brief low-luminance questionnaire was chosen due to the extensive nature of the functional testing and multimodal imaging protocol for the study. However, exploration of the utility of the NVQ-10 within the context of intermediate AMD recently conducted by our group suggested that its brevity may contribute to suboptimal performance.^37^ In addition, NVQ-10 scores reflect visual function in binocular conditions, whereas the other clinical measures represent uniocular visual function. Therefore, the non–study eye may have attenuated estimates of association between NVQ-10 and visual function. Questionnaire responses are subject to recall bias, and differences in lived experience may lead to divergent levels of self-reported functional vision for individuals with the same functional capabilities. In addition, ceiling effects were apparent among this cohort of participants with relatively good visual function. It is possible that a stronger relationship between NVQ-10 and DACP parameters may be detectable among people with greater levels of visual impairment. Notwithstanding, we found a weak-to-moderate correlation between NVQ-10 scores and RIT.

The current study has a relatively small sample; however, despite this we did observe significant associations between RIT and other clinical measures of visual function. Additional participants with a range of phenotypes typical of early and intermediate AMD would have allowed a more detailed investigation into the modifying effect of disease status on the relationship between DACP and the other parameters. Furthermore, a larger sample size may have revealed evidence of associations between the cyan-red sensitivity difference and the visual acuity–based tests that were reported as inconclusive in this study.

Strengths of our study included the systematic and consistent testing methods and data collection, detailed retinal grading, disease classification based on multimodal imaging, and the inclusion of control participants. In addition, sensitivity was tested at multiple retinal loci, and modern statistical approaches were employed to provide valid estimates of association.

### Biological Mechanisms

The reduction of retinal sensitivity in the central visual field of people with AMD is consistent with the known pattern of rod cell loss, which is greatest in the parafoveal region.^38^^,^^39^ BCVA, LLVA, and NVQ-10 are measures of cone function but also require parafoveal involvement. We hypothesize that a subtle decline of visual acuity, in the magnitude of letters rather than lines, may occur early in the AMD process and that the differences in function detected in this study are indicative of dysfunction in both rod and cone cells.

### Future Research

Our group has previously reported on changes in rod sensitivity over a 12-month period among people with intermediate AMD and controls.^30^ However, during that study period, the clinical AMD status of participants did not change (i.e., none of the participants progressed from control to AMD or from intermediate to late AMD). Prolonged follow-up of a larger sample is required to assess the covariance between measures of visual function and anatomical markers of disease progression.

Currently available low-luminance questionnaires often display ceiling effects in the absence of late AMD.^12^^,^^36^ A more robust instrument for capturing self-reported low-luminance visual function would allow the relationship between retinal sensitivity and functional vision to be investigated in more detail.^40^ In addition, assessing both eyes of each participant via DACP will allow a more valid comparison with vision-related quality-of-life instruments that relate to binocular function.

## Conclusions

In this cross-sectional study, we found that RIT was associated with BCVA, LLVA, and NVQ-10 score. A decrease in rod function in the early stages of AMD may be indicative of more generalized photoreceptor dysfunction, involving cones as well rods. Further work on questionnaires that specifically target visual function in dim and dark conditions may provide an easy to administer test that provides useful data on rod function without the need to perform more difficult perimetric tests of rod function.

## Supplementary Material

Supplement 1
