# Supplementary material for: Relationship Between Rod-Mediated Sensitivity, Low-Luminance Visual Acuity, and Night Vision Questionnaire in Age-Related Macular Degeneration
Source: Transl Vis Sci Technol. 2020 May 28;9(6):30. doi: 10.1167/tvst.9.6.30 (PMC7409161; doi:10.1167/tvst.9.6.30)
Supplement: Supplement 1 [file tvst-9-6-30_s001.pdf]

Supplementary material

Relationship between rod-mediated function, low luminance visual acuity and Night Vision Questionnaire in age-related macular degeneration

Stata statistical computing code to estimate the rod intercept time (RIT) for each test locus

```
gen rit = .
label var rit "Rod intercept time (minutes)"

label define error_rit_ 1 "> 30 mins" 2 "<= 0 mins"   ///
      3 "Observed log(cd/m2) too high" 4 "Observed log cd/m2 too low"
gen error_rit = .
label var error_rit "Undefined rod intercept time"
label values error_rit error_rit_

local list4 "-90 0 90 180"          // Meridians for points at 4 degrees
local list5 "-135 -45 45 135"       // Meridians for points at 5.657 degrees
local list8 "-90 0 90 180"          // Meridians for points at 8 degrees
foreach i of numlist 1/98      {    // Participant ID
foreach r of numlist 4 5.657 8 {    // Ring
      if `r' == 5.657 {
            local rl = 5            // Can't have decimals in name of local macro
      }
      else {
            local rl = `r'
      }
foreach m of numlist `list`rl'' {   // Meridian

      disp _newline "ID `i', Ring `r', Meridian `m'."
      local if "if id == `i' & ring == `r' & meridian == `m'"

noisily capture {

      quietly nl  ///
      (log_c = log10(10^({Tf} + {Ti} - ({R} *(time_m))) + 10^{Tf})) `if', nolog

      matrix A = r(table)
            local Tf = A[1,1]                   // Final threshold
            local Ti = A[1,2]                   // Initial threshold
            local R  = A[1,3]                   // Rate of decay
```

```
	capture drop pred_luminance
	quietly predict pred_luminance `if'	// Predicted luminance at each time

	*	Estimate RIT (in minutes) (Time when luminance = -3cd/m3)
	replace rit = (log10((10^-3) - (10^`Tf')) - `Tf' - `Ti') / - `R' `if'

	*	Signal error if function doesn't cross -3 cd/m2
	foreach v of varlist pred_luminance log_c {
		sum `v' `if', meanonly
			local max_`v' = r(max)
			local min_`v' = r(min)
	}
	`if' & (`max_pred_luminance' < -3) & (`max_log_c' < -3)  {
		replace error_rit = 4 `if'
	}	// log cd/m2 too low
	`if' & (`min_ pred_luminance' > -3) & (`min_log_c' > -3) {
		replace error_rit = 3 `if'
	}	// log cd/m2 too high

}		//   capture
}		// m Meridian
}		// r Ring
}		// i Participant ID

*	Exclusion criteria
replace error_rit = 1 if rit > 30 & rit != .
replace error_rit = 2 if rit <= 0
```

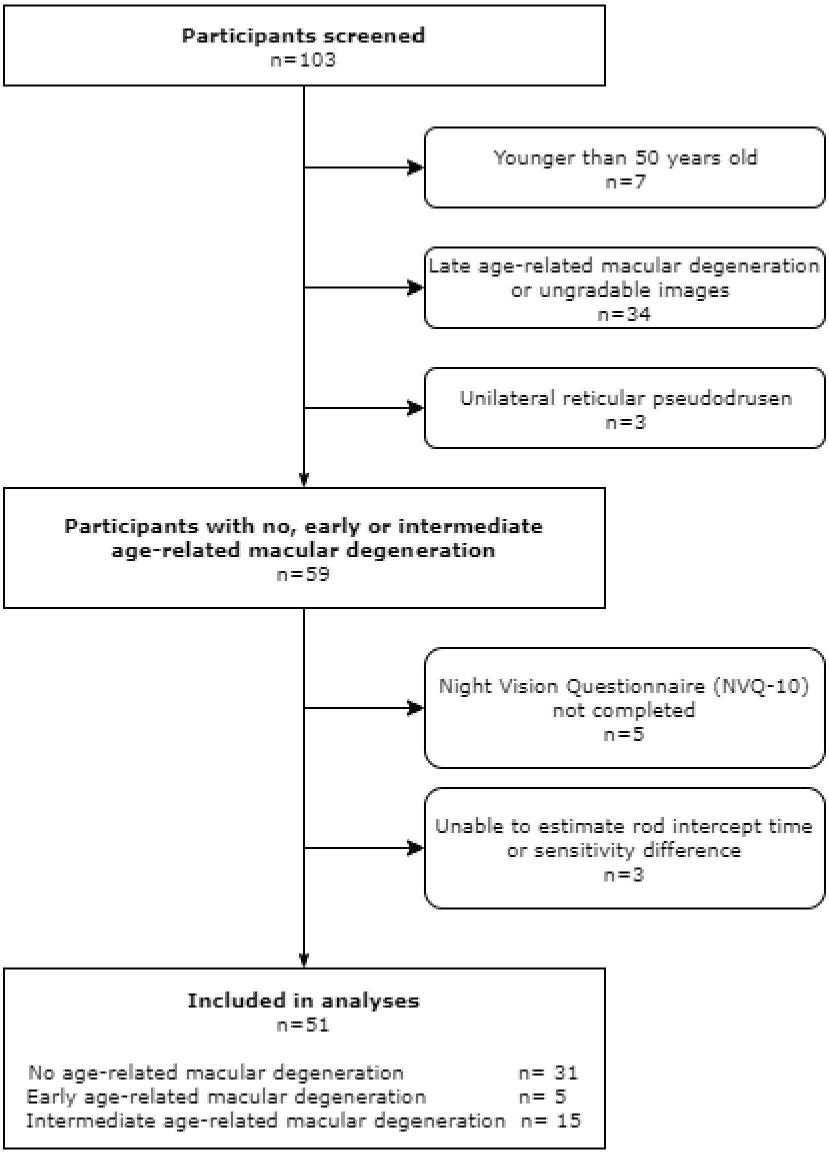

Supplementary Figure 1: Participant flow chart

Supplementary Table 1: Percentage of responses for each category of the ten-item Night Vision Questionnaire (NVQ-10). n=51

Item	Question	0 Stopped doing this because of your eyesight	1 Extreme difficulty	2 Moderate difficulty	3 A little difficulty	4 No difficulty at all	Not applicable*
1	How difficult is it for you to see moving objects, such as people or other cars when driving at night? Would you say you have:	2.0	0.0	0.0	2.0	86.3	9.8
2	How difficult do oncoming headlights or streetlights make it for you to drive at night? Would you say you have:	2.0	0.0	7.8	13.7	66.7	9.8
3	How difficult is it for you to read street signs when driving at night? Would you say you have:	2.0	3.9	5.9	7.8	70.6	9.8
4	How difficult is it for you to see street signs when you are a passenger in the car at night? Would you say you have:	0.0	2.0	9.8	0.0	86.3	2.0
	**Tell us how bothered you are by these items**	**0 Very bothered**	**1 Somewhat bothered**	**2 A little bothered**	**3 Not bothered at all**		
5	Poor vision at night	0.0	3.9	7.8	88.2		
6	Problems in reading in dim light	11.8	9.8	21.6	56.9		
7	A dark spot in the middle of my vision in dim light	2.0	0.0	0.0	98.0		
8	Poor vision in dim lighting	0.0	3.9	9.8	86.3		
9	Problems adjusting to the dark when entering a theatre	0.0	11.8	5.9	82.4		
10	Trouble seeing the stars in the sky at night	0.0	2.0	2.0	96.1		

*This response options *Not currently driving* and *Stopped doing this for other reasons* were treated as missing for the purposes of this analysis
Empty cells indicate that the response was not available for that item. Response options chosen by no participants shaded in gray.
